# Network dynamics reveal drought synchronization hubs in the Po River Basin

**DOI:** 10.1038/s41598-025-02878-z

**Published:** 2025-08-08

**Authors:** Antonio Zinilli, Edmondo Di Giuseppe, Arianna Di Paola, Sara Quaresima, Massimiliano Pasqui

**Affiliations:** 1 Research Institute on Sustainable Economic Growth, National Research Council, Rome, Italy; 2 Institute of BioEconomy, National Research Council, Rome, Italy

**Keywords:** Complex networks, Climate sciences, Hydrology, Physics, Statistical physics, thermodynamics and nonlinear dynamics

## Abstract

The intensifying climate crisis has exacerbated the frequency and severity of prolonged droughts, particularly in environmentally and socio-economically vulnerable climate change hot-spot regions. Despite advancements in monitoring, the spatiotemporal propagation and interdependencies of drought events remain poorly understood. This study analyzes drought synchronization within the Po River Basin, a critical hydrological system contributing approximately 40% of Italy’s GDP. Using the 12-month Standardized Precipitation Index (SPI-12) and complex network methods, we reveal the spatiotemporal dynamics of drought propagation, identifying key hubs and hot-spot regions. Our analysis identifies spatial hubs where droughts originate and terminal zones where impacts converge. This process follows a diffusive propagation mechanism, whereby local events spread through preferential pathways until they are interrupted by seasonal climatic conditions that restore precipitation regime. These findings enhance understanding of drought dynamics in interconnected systems, advancing the application of complex network theory to hydro-climatology. They also provide a foundation for research on societal resilience to climate change and the development of adaptive strategies for sustainable hydrological systems.

## Introduction

Drought can be defined as an extended period of abnormally low precipitation^1,2^ often resulting in water shortages, potentially adversely affecting agriculture, ecosystems, and society^3–5^. The effects of drought are often exacerbated by extreme heat, high rates of evaporation, and human water usage^1,6,7^. Additionally, climate change is increasing the frequency and severity of drought events, leading to more prolonged and widespread dry periods^8–12^. Drought conditions evolve temporally and spatially, affecting different components of the water cycle^13^, which adds complexity to understanding and managing the phenomenon. Assessing drought propagation remains a complex challenge that requires deeper investigation to improve planning resilience strategies to cope with climate variability and change and to support more effective drought management strategies^14–16^. Additionally, drought propagation itself is inherently complex due to its spatiotemporal variability and the cascading nature of impacts across interconnected regions.

In this paper, we aim to address this challenge by studying drought through a complex network approach, focusing on the synchronization of drought events. Complex network examines how droughts spread and interact across regions, offering a broader view of the drought propagation and identifying emerging structures that could cover large areas and form spatial clusters. In this context, network complexity stems from its non-trivial topology, characterized by heterogeneous connectivity distributions, modularity, and the presence of pathways that influence the spread of drought events. Specifically, we investigate the network of sub-regions particularly exposed to episodes of drought within the Po River Basin, Italy’s largest river basin. Its agricultural and industrial significance makes it a critical area for understanding drought impacts, and in recent years, the basin has experienced multiple drought events, raising national concerns over the increasing frequency of droughts and future water resource availability^17–20^.

Over the past decades, numerous drought indices have been developed to monitor and assess drought conditions^1,6,20–22^. Among these, the Standardized Precipitation Index (SPI), introduced by McKee et al.^23^ has become one of the most widely adopted tools for quantitative drought assessment. Its popularity stems from its ease of calculation, its ability to represent meteorological and climate conditions across different timescales and locations, and its interpretability in probabilistic terms with respect to impacts. This makes it suitable for spatial comparisons and operational decision-making at local and regional levels. The SPI calculation, as proposed by McKee^23,24^, incorporates temporal scales to define the duration over which precipitation anomalies are aggregated and analyzed. A 12-month timescale (SPI-12), for instance, standardizes annual precipitation, making it ideal for long-term hydrological drought monitoring by minimizing seasonal fluctuations and capturing precipitation deficits significant enough to alter water availability across the river basin.

Given the complexity and multifaceted impacts of drought, no universal method currently exists for assessing drought comprehensively^1^. While evidence highlights the increasing frequency and severity of droughts in the Po River Basin^17,19,20^, the spatiotemporal dynamics of drought propagation, and their connection to climate anomalies, remain insufficiently understood. Advancing societal resilience to the climate crisis requires a deeper integration of drought-related scientific knowledge into effective and adaptive policy development^25^. This gap is partly due to the tendency of existing studies to primarily focus on localized analyses, often overlooking the broader networked interdependencies that drive drought synchronization. By integrating conventional drought metrics-such as intensity, duration, and frequency-with a complex network-based framework, this study provides a novel perspective on drought propagation patterns, emphasizing the dominant pathways and revealing critical drought hubs within the basin. Additionally, recent developments in complex network theory offer a flexible and robust analytical framework for capturing the temporal and spatial interdependencies of drought events, applicable across multiple spatial scales-from global to local contexts^26^. This approach reveals underlying patterns in the spread of droughts from one region to another, offering insights that enhance our ability to understand and predict possible evolution, increasing the formulation capabilities of coping options^27–29^. A key indicator of systemic vulnerability in complex networks is the propensity for localized events to propagate across the network, creating prevalent direction of drought diffusion through sync-coherent community of nodes, while affecting distant, interconnected areas. In this framework, the impact of different synchronised droughts episodes occurring over different sub-regions may amplify substantially the level of complexity of managing drought impacts at larger scale^30^. Network theory provides a robust framework for identifying and mapping these processes, offering insights into how events synchronize, propagate and interact across spatial and temporal scales. To date, significant knowledge gaps remain in understanding the spatial interconnections, clustering patterns and propagation pathways of drought events, particularly in complex hydrological systems such as the Po River Basin. Addressing these gaps requires a systematic exploration of drought dynamics using network-based approaches.

In this study, we aim to answer the following research questions: (a) How are drought events interconnected within the Po River Basin? (b) Are there key areas that share similar propagation dynamics during major drought episodes? (c) How might these key-areas reflect distinct functional roles within the basin-such as initiation zones, transit corridors, or accumulation sinks?

To address these questions, we analyze drought event synchronization within the Po River Basin using the SPI-12 index. We define a drought event as any period during which SPI-12 remains below a critical threshold for at least three consecutive months. Two events are considered synchronized if they meet this condition within a maximum window of three months, allowing us to capture patterns of drought occurrence and propagation across the basin. Our network-based approach focuses primarily on the Po River Basin but extends to surrounding areas to identify potential interconnected regions while keeping interpretations within the basin itself. This allows us to identify areas that serve as hubs for drought synchronization and comprehend the physical mechanisms underlying these relationships. The resulting outcomes offer a novel lens through which to interpret basin-wide drought dynamics, potentially guiding decision-makers in prioritizing areas for enhanced monitoring and intervention.

### Problem formulation

This study explores the synchronization of drought events in the Po River Basin through a network-based approach, examining both undirected and directed network structures. By analyzing interactions between a number of locations (nodes) represented by ECMWF Reanalysis v5 (ERA5) grid points^31^, we assess how connections within and around the basin influence drought synchronization over time.

Specifically, given a pair of nodes $i$ and $j$, the network-based approach quantifies the frequency of synchronized drought events between them during the period 1980/2023. To achieve this, we constructed an undirected graph $G(N, L)$, where nodes (N) represent locations and links (L) indicate synchronized drought events over the study period. This undirected network allows for the analysis of synchronization probabilities and the identification of connectivity patterns across the basin. Building on this foundation, a directed network approach is introduced to analyze the directional propagation of drought events. In the directed network, links represent the occurrence of drought events from one location to another, offering insights into dynamic patterns of propagation.

**Fig. 1:**
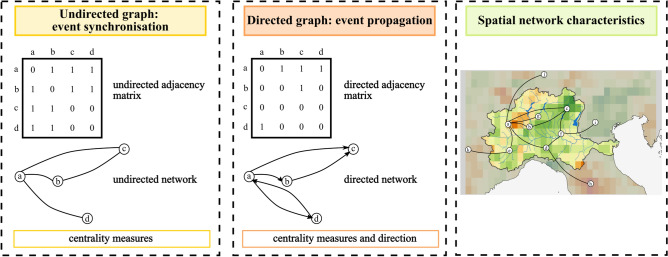
Representation of undirected and directed complex networks.

Figure 1 provides a visual guide to these analyses. Panel 1 presents the undirected network, including its adjacency matrix and the network structure, focusing on overall synchronization. Panel 2 illustrates the directed network, emphasizing event propagation. Panel 3 integrates these network types with the basin’s geography. The undirected network captures overall synchronization patterns, while the directed network highlights the directionality of drought events propagation.

In the undirected graph $G(N, L)$ only inter-node links are considered, excluding loops. The primary objective of this analysis is to evaluate the probability of synchronization across the network based on historical data. Figure 3 summarizes these approaches and maps network characteristics to the basin.

Using complex network metrics, we quantify synchronization for each node in relation to all other nodes in the network. For the undirected network, we calculate metrics such as degree centrality, closeness centrality, and betweenness centrality. Degree centrality measures the number of direct connections a node has, identifying local synchronization hubs where drought events co-occur and influence neighboring regions. Closeness centrality quantifies how efficiently a node can reach all others in the network, traditionally reflecting its role in accelerating global synchronization by enabling the rapid spread of events. However, in the case of drought — an inherently passive phenomenon — high closeness centrality may instead indicate a node’s propensity to be rapidly reached by drought signals propagating through the system, highlighting its exposure rather than its role in transmission. Betweenness centrality highlights nodes that act as bridges between different parts of the network, facilitating drought propagation across otherwise weakly connected areas. While high-degree nodes contribute to widespread exposure, facilitating localized drought synchronization, closeness centrality highlights the speed at which a region can be affected by upstream drought effects. In contrast, high betweenness suggests that drought follows specific pathways rather than spreading uniformly across the network^32–34^. These measures provide insights into the structural importance of each node and the overall connectivity of the network, without accounting for the direction of event propagation. Since the undirected network does not include directionality, it reflects only the presence of synchronization between two nodes, not the direction in which drought event propagation occurs. In contrast, the directed network captures the directionality of drought event propagation, enabling an analysis of not only whether two locations are synchronized but also the propagation of drought events between them. For the directed network, we calculate metrics such as in-degree and out-degree centrality. These metrics identify nodes that act as sources of drought propagation (high out-strength) or as sinks that receive and accumulate drought events (high in-strength). Building on this analysis, we apply community detection to the undirected network using the Louvain method. This approach is particularly useful for identifying clusters of nodes that are more densely linked to one another than to the rest of the network, allowing us to determine groups of areas that tend to experience droughts in a coordinated manner. The Louvain method optimizes modularity, a measure of how well the network is divided into communities by maximizing connections within communities while minimizing those between communities^35^. It begins with each node as an independent community and progressively merges nodes into larger groups to maximize internal connections while minimizing external ones. The process consists of two main phases: first, a local optimization step, where nodes are reassigned to different communities to increase modularity; second, a hierarchical aggregation step, where the identified communities are collapsed into single nodes, and the process repeats at this higher level. This iterative approach continues until no further modularity improvements could be achieved, resulting in a hierarchical community structure that effectively captures the network’s organization^36^. To ensure the robustness of the community detection results and assess the stability of the detected community structure, we performed 1,000 independent runs of the algorithm. We then computed the Normalized Mutual Information (NMI) score for each pair of runs to quantify the consistency of the detected communities^37^. The results show an average NMI score of 0.88, indicating a high level of stability in the clustering structure across different runs. This high NMI score confirms that the three detected clusters remain consistent, despite the inherent stochasticity of the Louvain method. While small variations can occur, particularly for nodes at the boundaries of clusters (geographic outliers), the core community structure remains stable, reinforcing the reliability of the detected synchronization patterns. By applying Louvain community detection to the undirected network, we gain deeper insights into which areas are more likely to synchronize drought occurrences, complementing the directed network’s focus on propagation dynamics.

## Results

**Fig. 2:**
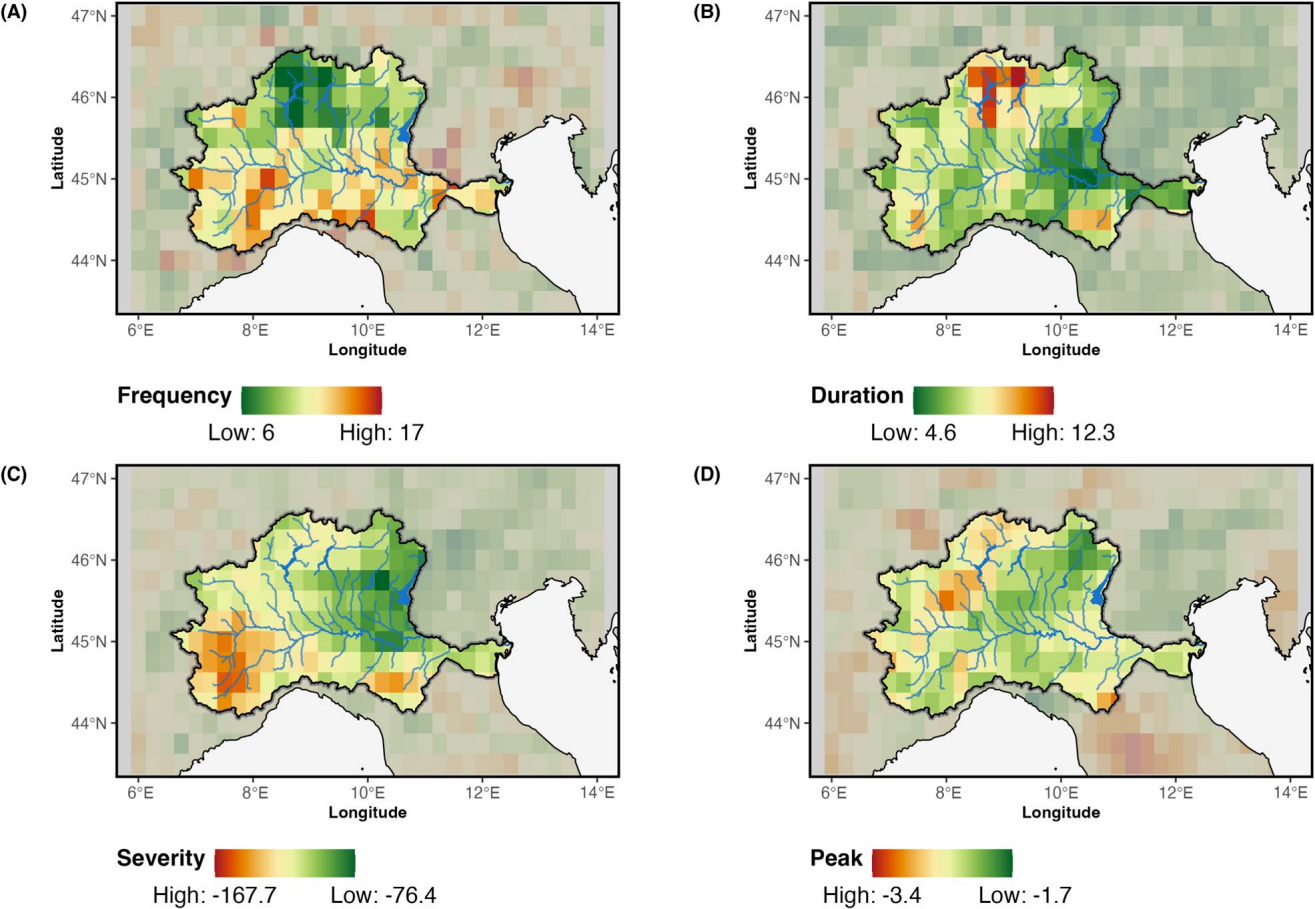
Spatial distribution of drought indicators. (**A**) the total number of drought events, (**B**) the average duration, (**C**) the total severity, and (**D**) the peak.

A joint analysis of conventional drought characteristics and a network-based approach provides a comprehensive understanding of drought dynamics, advancing our insight into the underlying mechanisms that shape the observed patterns. In this study, we first use established indicators, specifically focusing on drought frequency (Fig. 2a), duration (Fig. 2b), intensity (Fig. 2c), and peaks (Fig. 2d) derived from the SPI-12 to identify the most critical hydrological drought areas within the Po River Basin. These indicators assess each location (or grid point) independently, without accounting for connections or interactions between regions.

Figure 2a indicates that drought episodes are notably more frequent in the southern part of the Po River Basin. In contrast, Fig. 2b shows that drought events in the northern region tend to persist longer, with durations averaging up to 12 consecutive months. Figure 2c highlights that the severity of droughts is more pronounced in the western part of the basin, where extreme peaks are concentrated in the northwestern section. Lastly, Fig. 2d reveals that small areas in the southeastern, southwestern and northwestern parts of the basin faces particularly critical conditions, displaying a few grid points with high values of duration, severity, and peak intensity, despite experiencing fewer drought events overall.

**Fig. 3:**
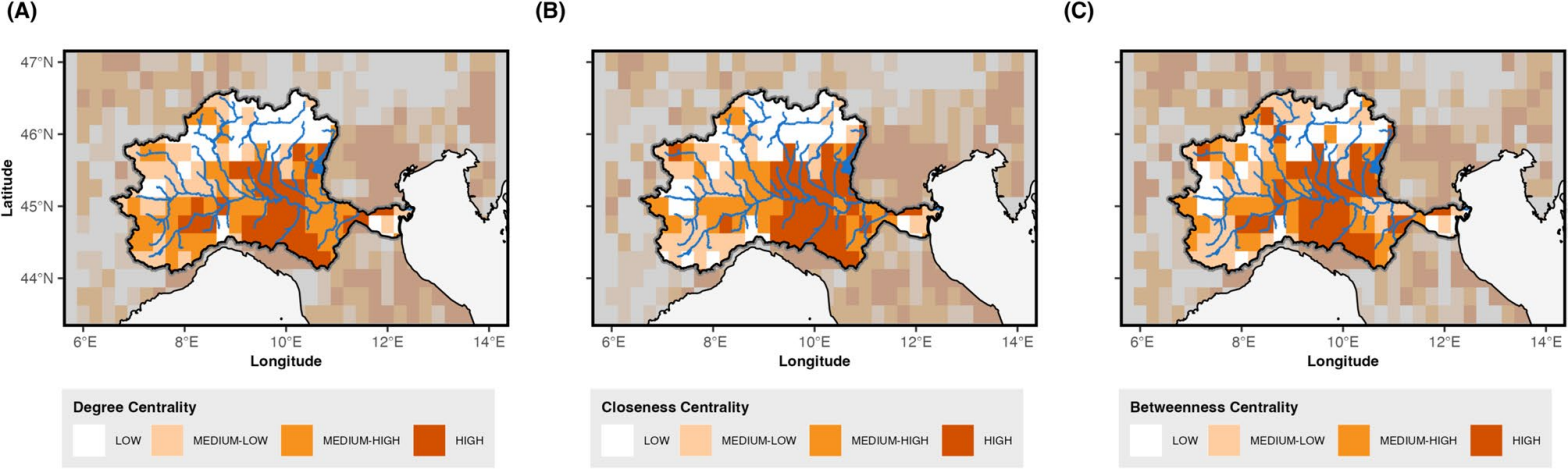
Centrality measures. (**A**) DC = Degree Centrality, (**B**) CC = Closeness Centrality, (**C**) BC = Betweenness Centrality. Centrality values are divided into four classes based on quartile ranges for better visualization: Low = 0-25th percentiles, medium-low = 25-50th percentiles, medium-high 50-75th percentiles, high $>=$ 75th percentile.

**Fig. 4:**
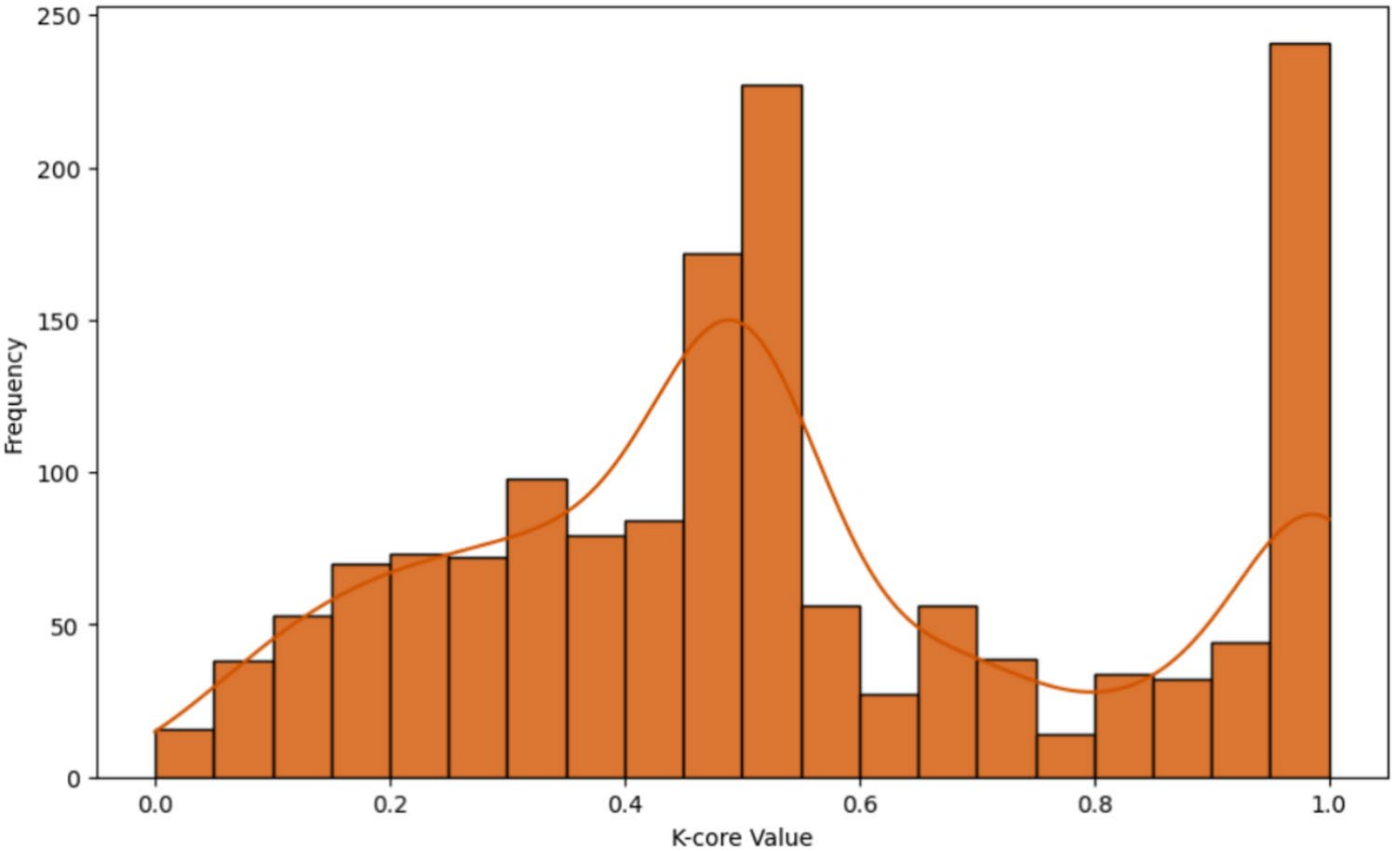
Distribution of K-core values.

**Fig. 5:**
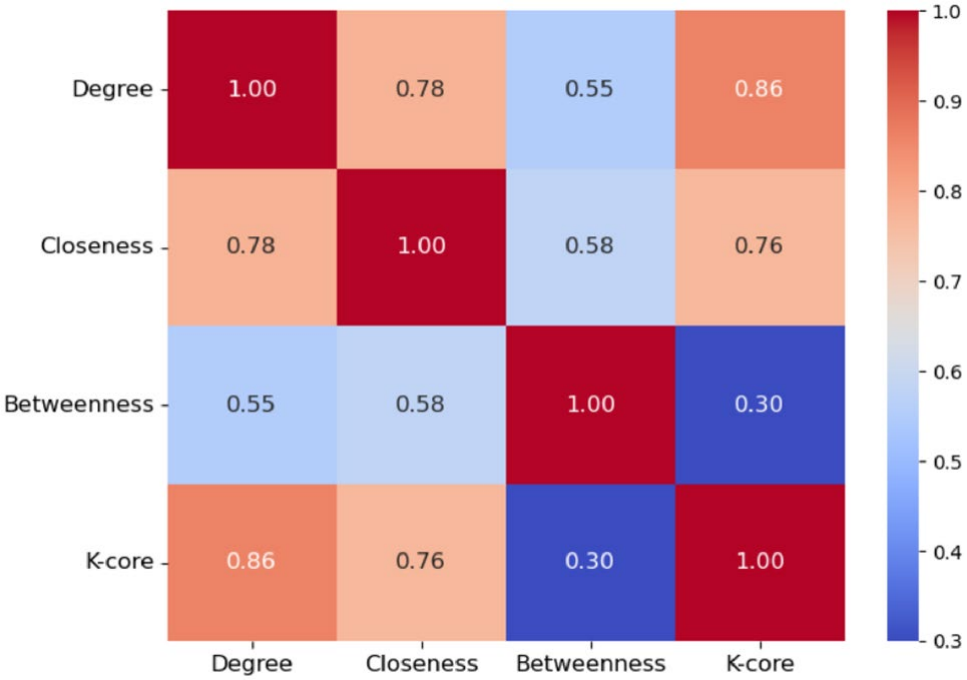
Correlation heatmap among centrality measures.

**Fig. 6:**
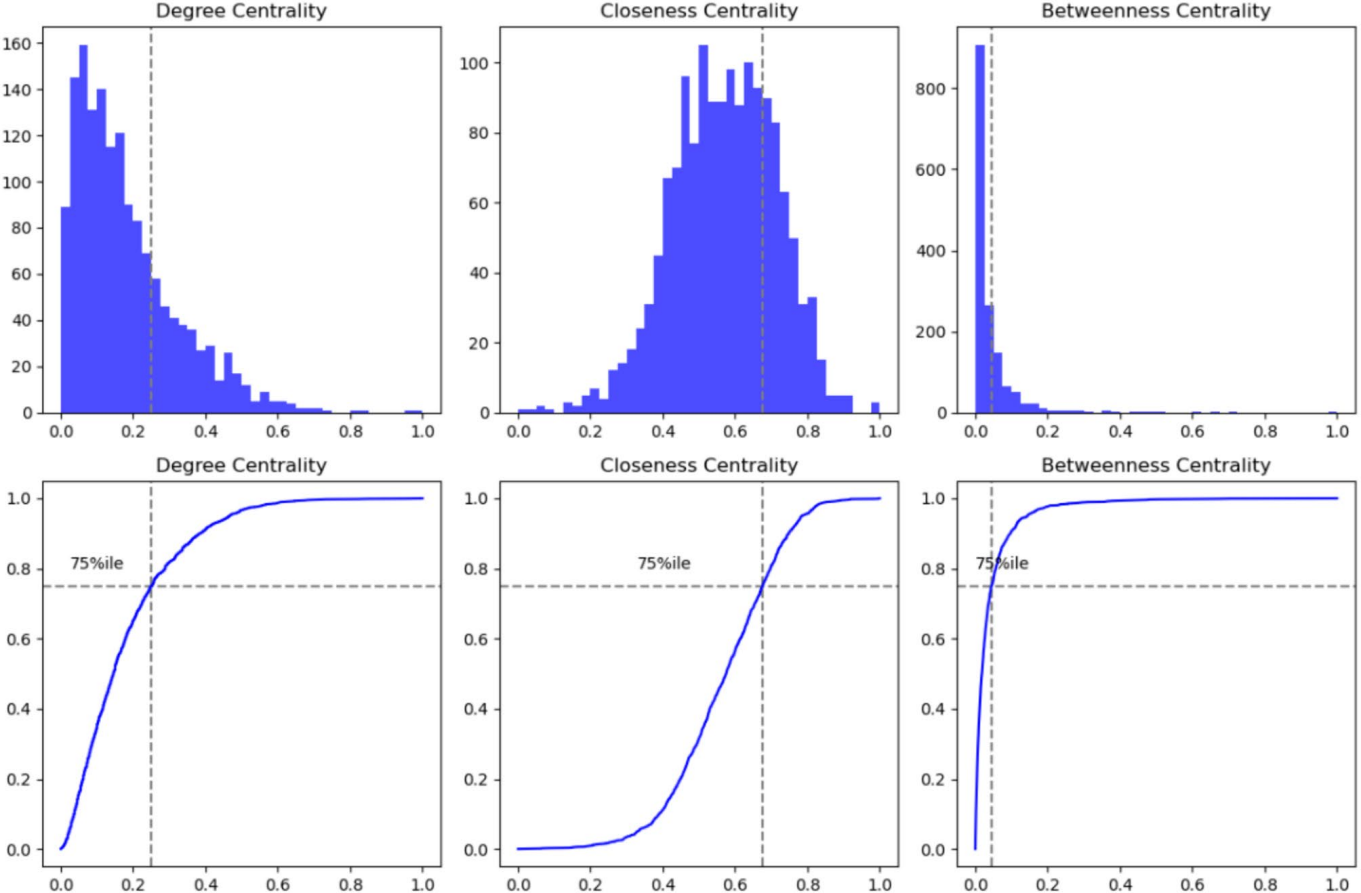
Distribution of centrality measures within the Po River basin. Upper panels: histogram; bottom panels: Empirical Cumulative Distribution Function (ECDF).

**Fig. 7:**
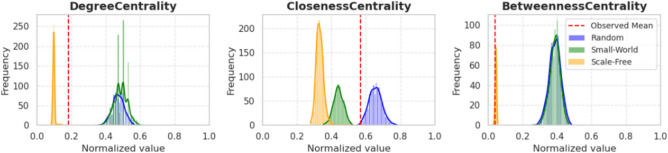
Centrality measures in the drought synchronization network vs. simulated models.

Overall, the spatial patterns indicate distinct regional zones of precipitation deficit within the Po River Basin, closely linked to the climate regimes outlined in Fig. 13. The southern region experiences more frequent yet shorter drought events, while the northern areas are characterized by more prolonged episodes. Additionally, the western sector and a relatively small portion of the extreme southeast display the most severe drought conditions. Building on these findings, we shift our focus to a network-based analysis in order to evaluate drought hot-spots not merely as isolated regions, but as interconnected nodes embedded within a broader system. Figures 3, 4, 5, 6 and 7 along with Table 1, expand the analysis of the structural role of regions within the basin’s drought network. Figure 3 presents the spatial distribution of centrality measures, categorized into four percentiles (>25th, 25-50th, 50-75th, >75th), offering a detailed view of the regions’ relative importance in the network. Figure 4 introduces the distribution of K-core values, highlighting the structural embedding of nodes within the network. The inclusion of K-core analysis allows us to differentiate between highly interconnected regions, where drought conditions may persist and reinforce, and more peripheral areas that are weakly embedded in the network. The correlation between centrality measures and K-core is then shown in Fig. 5 and Table 1. Complementing this, Fig. 6 displays histograms and empirical cumulative distribution functions (ECDF) for each centrality measure, summarizing the statistical properties of these measures across the study region. To determine whether the observed patterns reflect meaningful structural features or are instead driven by the inherent properties of the centrality measures, in Fig. 7 we compare the empirical network to a set of synthetic benchmark models-including random, small-world, and scale-free networks^38–40^. These null models offer a reference framework that helps isolate signatures of drought-related dynamics from structural patterns that may emerge purely from the network’s size or density.

Degree Centrality (DC) represents the number of connections each node (or grid point) has with other nodes in the network. Areas with a higher Degree Centrality (shown in darker shades of orange) are crucial hubs of network interaction. These regions are highly interconnected, indicating that drought events are likely to be synchronized with multiple other locations. Areas with a high Degree Centrality, particularly those located in the central and southeastern parts of the basin, act as local synchronization hubs, where drought conditions emerging in one area are more likely to co-occur with multiple nearby sub-regions. The southwestern region shows an intermediate DC, while the northern region shows no relevant DC, except in the two Alpine areas of the National Park of Gran Paradiso and Val Grande (the western side of Lake Maggiore).

The second map highlights Closeness Centrality (CC), which reflects how quickly a node can be reached by all other nodes in the network^34^. In the context of drought synchronization, areas with high Closeness Centrality (depicted in darker orange) represent regions that are, on average, closer to other areas in terms of connectivity and thus easily accessible. This network perspective highlights them as key nodes for understanding how drought signals travel across the system and for implementing effective early warning strategies, as their central position makes them more likely to experience drought conditions in synchrony with other sub-regions across the basin. Similarly to DC, the southwestern region shows an intermediate level of CC while the northern region shows no relevant CC but an intermediate level in the Gran Paradiso and Val Grande areas. The third map highlights Betweenness Centrality (BC), which measures the extent to which a node lies on the shortest path between other nodes. Regions with high Betweenness Centrality (shown in darker shades of orange) act as crucial pathways in the network, facilitating the transmission of drought conditions between different areas. These regions do not necessarily experience the most severe droughts themselves, but they play a key role in channeling the propagation of drought events along preferential routes, influencing how and where the drought spreads across the basin. Again, the southwestern region shows an intermediate level of BC, while the northern region shows no relevant BC but a high level in the Gran Paradiso and Val Grande areas.

When these three centrality measures are analyzed together, clear correlations emerge between them, as observed in their spatial distribution across the maps. Regions that exhibit high values across all three measures are likely to experience synchronized drought events. These areas not only have numerous connections (high DC) but are also characterized by low average distances to all other nodes in the network (high CC) and serve as bridges for drought propagation (high BC). To further investigate the structural role of these highly central regions, it is essential to consider the underlying network topology. The K-core analysis provides a way to differentiate between structurally embedded nodes and those at the periphery, revealing the hierarchical organization of the network^41^. Higher K-core values indicate nodes that belong to a densely connected core, which likely plays a fundamental role in drought propagation. The distribution of K-core values is shown in Fig. 4, which provides more information about the network’s cohesiveness. Spatial and topological restrictions frequently cause the correlation between centrality measures like Degree, Closeness, and Betweenness to emerge in geographically limited systems.

Figure 4 reveals that the network does not exhibit a strict core-periphery structure, but rather a continuous connectivity pattern. The bimodal distribution suggests the presence of a group of regions with intermediate K-core values (0.45–0.55) and a more structurally embedded group with high K-core values. This pattern aligns with the nature of climate-driven phenomena, where drought synchronization is shaped by spatially and temporally interconnected processes rather than discrete structural boundaries. To better understand the relationships between different centrality measures, we computed the correlation matrix between Degree, Closeness, Betweenness, and K-core. Figure 5 presents a heatmap of the correlation coefficients, while Table 1 reports the corresponding descriptive statistics for each measure.

**Table 1:** Descriptive statistics for Degree, Closeness, Betweenness and K-core measures.

Statistic	Degree	Closeness	Betweenness	K-core
Count	1525	1525	1525	1525
Mean	0.18	0.57	0.04	0.54
Std. Dev.	0.14	0.14	0.07	0.28
Min	0	0	0	0
25%	0.07	0.47	0.01	0.33
Median	0.14	0.57	0.02	0.51
75%	0.25	0.67	0.04	0.71
Max	1	1	1	1

Figure 5 and Table 1 provide a joint view of the correlation matrix among centrality measures and their descriptive statistics. The correlation analysis reveals a moderate relationship between Betweenness centrality and Degree (r = 0.55), as well as between Betweenness and Closeness (r = 0.58). In networks where the propagation of events is dominated by a few critical nodes, stronger correlations would be expected. Betweenness appears more evenly distributed across multiple nodes, as confirmed by the descriptive statistics in Fig. 5. The median value is only 0.02, and the interquartile range is narrow, indicating that a large share of nodes exhibit low Betweenness values. This indicates that while a few nodes may be key facilitators of drought event synchronization, many others contribute only marginally. Unlike Betweenness, Degree and Closeness exhibit higher average values (0.18 and 0.57, respectively), reflecting more consistent local connectivity and proximity across the network. The role of the K-core structure further supports this interpretation. The strong correlation between K-core and Degree (r = 0.86), and its reduced correlation with Closeness (r = 0.76), suggest that structural embeddedness contributes significantly to synchronization processes. In contrast, the low correlation between K-core and Betweenness (r = 0.30) indicates that densely interconnected regions are not necessarily the key intermediaries in event propagation. The K-core measure, with a mean of 0.54 and an interquartile range between 0.33 and 0.71, highlights a moderate level of embedding for most nodes. Taken together, these results suggest that drought synchronization across the network is sustained by a combination of structurally embedded cores and a small subset of nodes with high Betweenness that act as key intermediaries. However, the majority of nodes exhibit low Betweenness values, indicating that the propagation of drought events relies on both localized reinforcement within dense cores and a few strategic bridging nodes, rather than on a highly centralized structure dominated by hubs.

Degree, Closeness, and Betweenness centrality measures exhibit a heterogeneous distribution, as shown in the histogram and ECDF of Fig. 6. Degree centrality (DC) shows an asymmetric distribution skewed toward higher values. The ECDF indicates that the 75th percentile is close to 0.06, highlighting regions with significantly more connections than the rest. Betweenness centrality (BC) has a strongly right-skewed distribution, with most nodes having low BC values and only a few acting as critical intermediaries; notably, the 75th percentile (0.002) represents only a small portion of the total range $(0.0;\, 0.010)$. In contrast, Closeness centrality (CC) has a more uniform distribution compared to DC and BC. The ECDF shows the 75th percentile at approximately 0.45, indicating that most nodes have a relatively consistent capacity to synchronize with other nodes in the network, reflecting a uniform tendency to propagate or receive drought signals across the study area.

The distributions indicate that a few sub-regions act as critical hubs (high DC), synchronizing drought events locally, while others are strategic connectors (high BC), being on the fast-track propagation pathways across the network. Most areas exhibit similar capacities for the onset or receiving of drought event propagation (uniform CC). This highlights the importance of targeting key regions for effective drought management and mitigation.

To better understand whether the observed structural properties of the drought synchronization network are a consequence of domain-specific dynamics or inherent to the centrality metrics themselves, we compared the empirical distribution of centrality values (Degree, Closeness, and Betweenness) with those obtained from randomly generated networks. We employed three canonical network models — Random (Erdős-Rényi), Small-World (Watts-Strogatz), and Scale-Free (Barabási-Albert) — to generate ensembles of synthetic graphs that match the empirical network in size and density. The distributions for each centrality measure were normalized and plotted against the observed values represented by the red dotted line (Fig. 7).

The results reveal that while some differences may indeed stem from the mathematical properties of centrality measures, the comparison with null models allows us to distinguish topological effects from real dynamics. For example, the observed Betweenness Centrality distribution closely matches that of scale-free networks, suggesting the presence of hub-like nodes that mediate drought propagation across the network. This structure implies that a few critical areas may serve as nodes that mediate the synchronization of drought events across the network. On the other hand, Degree Centrality in the observed network is markedly lower than in Random and Small-World models but higher than in scale-free networks, suggesting a moderate level of local connectivity that is not solely attributable to a few dominant hubs. Finally, closeness centrality in the observed network exhibits higher values than in scale-free and Small-World networks. By comparing with synthetic benchmark networks, we disentangle the extent to which drought-related patterns are structurally meaningful, avoiding over-interpretation due to the intrinsic properties of centrality measures.

**Fig. 8:**
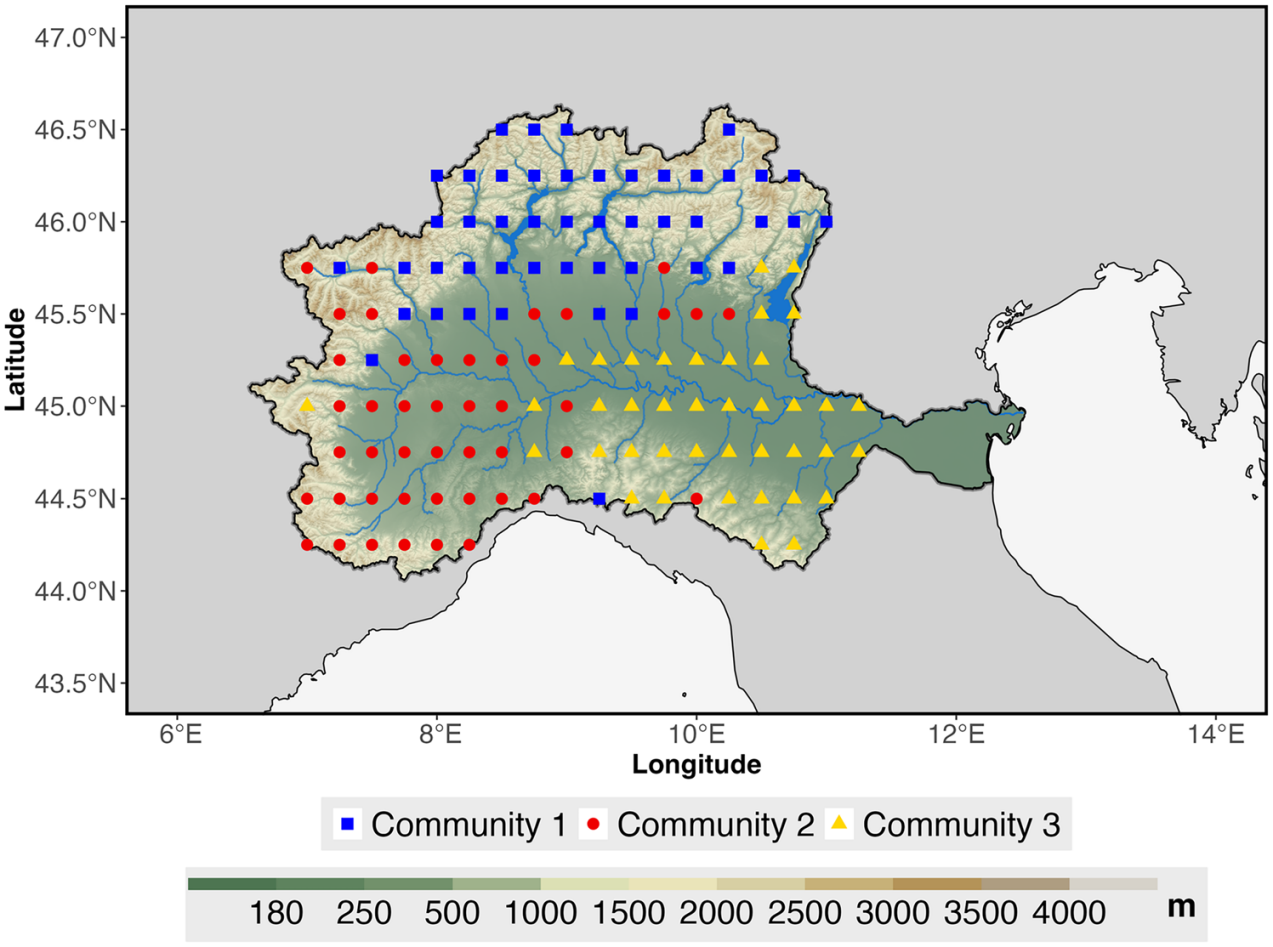
Network community detection applied to the Po River Basin.

We apply community detection to identify clusters of regions more closely connected within the network. This approach helps reveal how drought synchronization patterns are spatially organized within the basin. Figure 8 shows the results of the community detection analysis derived using the Louvain algorithm. Nodes are color-coded based on community membership: blue represents community 1, red indicates community 2, and yellow corresponds to community 3.

Community 1 (blue squares) is primarily located in the northern, high-altitude areas of the basin. Community 2 (red circles) shows a scattered distribution across the central region, with several overlapping zones at the boundaries of the other communities. Community 3 (yellow triangles) is mostly concentrated in the eastern part of the basin.

**Fig. 9:**
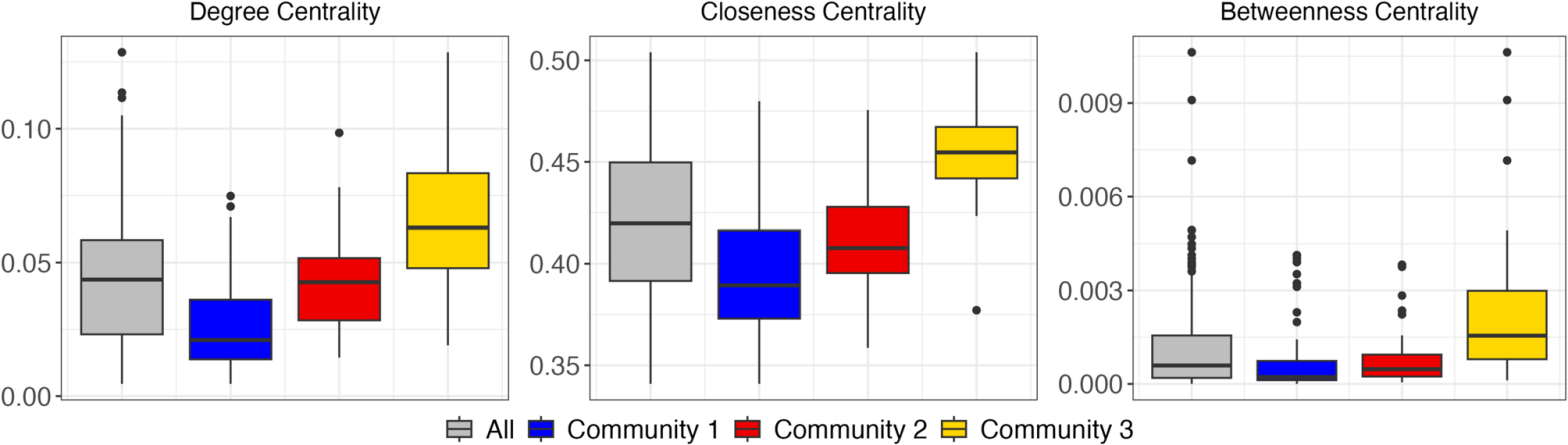
Distribution of centrality measures across the communities. Each box plot shows the median (central line), interquartile range (colored boxes) and full range (whiskers), with outliers represented by individual points beyond the whiskers. The grey box represents data for the entire Po River Basin, while the blue, red, and yellow boxes correspond to Community 1, Community 2 and Community 3, respectively.

**Fig. 10:**
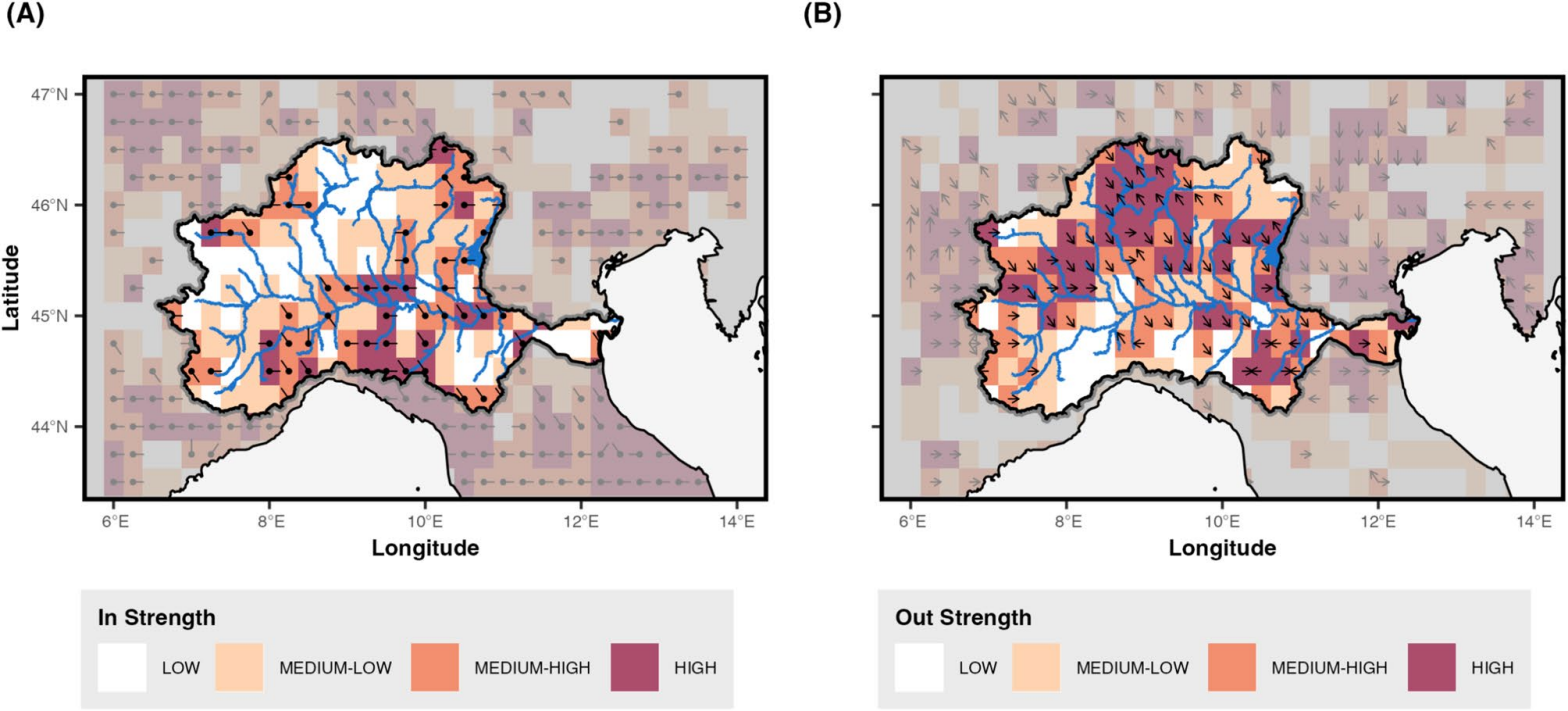
Directional network analysis. Spatial distribution of (**A**) In-strength and (**B**) Out-strength across the Po River Basin.

The in-depth analysis of network measures across the three identified communities reveals distinct structural and functional characteristics. The box plots in Fig. 9 illustrate the distribution of the three centrality measures within the identified communities, while Fig. 10 further highlights the spatial distribution of the In-Strength and Out-Strength derived from the directional network analysis.

The patterns indicate that Community 3 consists of regions with elevated drought synchronization, serving as hot-spots where drought events may have a large footprint in terms of area and point connections. In comparison, Community 1 shows lower connectivity, making these areas characterized by more localized drought events, while Community 2 represents an intermediate state between the two extremes.

Figure 10 shows the spatial distribution of In-Strength and Out-strength resulting from the directional network. The arrows depict the direction from which the drought events are entering (In-Strenght) or leaving (Out-Strenght). In the Out-strength map (right panel), the dark-shaded nodes identify locations where drought conditions initially arise and subsequently spread to neighboring areas (i.e., onset areas). Many of the onset areas are located in the central-northern part of the basin, with some positioned along the Alpine mountain arc.

The In-strength map (left panel) highlights nodes that frequently receive drought signals originating from other areas (darker shades). These regions represent points of propagation convergence, where drought conditions are more likely to be sustained by incoming signals rather than local initiation, suggesting a potential exposure to externally-driven hydrological drought impacts.

The regions identified with high Out-strength scores, particularly in the central and northeastern parts of the basin, function primarily as sources of drought events. These areas are key initiators of droughts, highlighting the onset of the spread of dry conditions towards other parts of the basin. Their role as sources implies that local environmental or climatic factors in these areas are conducive to the onset of droughts, which then propagate outward to affect broader regions.

The lower mean values in DC, CC and BC — measured on the undirected network — indicate that nodes within Community 1 have fewer connections, potentially reflecting regions within the basin that are more isolated or less integrated into the broader patterns of drought synchronization. However, the high Out-strength within Community 1 — measured on the directed network (see Fig. 10) — suggests that, despite their relative isolation, these regions serve as key sources of drought propagation, playing a crucial role in initiating or spreading drought conditions. This occurs because, in Community 1, nodes tend to have a low number of links compared to other nodes in the network, but these few links are mostly or entirely outgoing (high Out-Strength). Therefore, although these nodes are not highly connected in terms of overall network structure, they play a crucial role in initiating and spreading drought events to other regions.

In contrast, Community 3 displays the highest average values in all centrality measures (see Fig. 9) except for Out-strength (see Fig. 10), indicating an important role in the network but with a distinct capacity. The elevated Degree and Closeness Centrality values suggest that nodes in Community 3 are highly connected and central to the network structure, representing regions that are particularly prone to drought synchronization due to their prominence in the network dynamics. The elevated Betweenness Centrality in this community highlights its role as a major pathway for transmitting drought signals, connecting other regions within the network. The high In-Strength (Fig. 10) indicates that Community 3 is a primary recipient of drought regimes while also redistributing these conditions throughout the network, underscoring its dual exposure and influence within the system.

Community 2 occupies an intermediate position, with network measures generally falling between the corresponding values of Communities 1 and 3. This suggests that Community 2 exhibits moderate levels of drought proneness, influenced by characteristics shared both with Community 1 and Community 3, without displaying the extreme features of either.

Overall, these findings reveal a hierarchical structure within the network, with Community 3 emerging as the central hub for drought synchronization, Community 1 serving as a key region for propagating drought regimes, and Community 2 functioning as an intermediate area that mediates the propagation of drought dynamics across the basin. This hierarchical differentiation among the communities is significant for understanding and managing drought impacts in the Po River Basin, highlighting the need for targeted strategies that account for the varying roles of different regions in the network.

## Discussion

In this study, we investigated the synchronization of drought events across the Po River Basin using the Standardized Precipitation Index at 12 month-scale (SPI-12)—to account for significant and persistent drought processes that can impact the water availability at the river basin scale—and Complex Network analysis. By examining centrality measures (Degree, Closeness, and Betweenness) and applying community detection, we characterized each region’s role within the drought network. Additionally, In-Strength and Out-Strength metrics were employed to examine drought onset and propagation dynamics, shedding light on regional exposure to hydrological drought hazard and their interconnections within the basin.

Overall, the results identified three macro-areas (communities) within the basin, each characterized by distinct drought profiles. Each area plays a well-defined role within the network, which can be interpreted as follows. Community 1, covering the northern Alpine region, is characterized by low centrality values (DC, BC, CC), experiencing relatively few drought events, yet with longer durations and high Out-Strength. From a traditional drought perspective, this indicates that droughts in this area are less frequent but tend to be more persistent, increasing the region’s susceptibility to prolonged dry conditions (Fig. 2). This pattern is typical of mountainous and foothill zones, where orographic enhancement and convective activity usually ensure higher rainfall. However, such dependence on specific meteorological patterns makes these regions highly sensitive to climatic disruptions: shifts in low-pressure systems or reduced vertical instability can significantly diminish precipitation. When drought does occur, its impacts tend to be more severe, affecting both the local area and adjacent downstream regions. This behavior aligns with the network analysis results, which highlight a low degree of connectivity but a notable role as a source of drought propagation when events do occur. Acting as a natural barrier, the Alps exert a strong local dynamic footprint when interacting with large-scale atmospheric circulation across all seasons. In winter, snow droughts may produce long-term signature in the SPI-12 index^42^ resulting in prolonged drought events, while the mountain ridge may physically block incoming western and northwestern disturbances, inhibiting precipitation over the valley and contributing to a downstream propagation of drought conditions.

Community 3 is predominantly located in flatter regions and is characterized by typically short-lived drought events. In these areas, precipitation deficits tend to be less intense, partly due to the naturally drier baseline climate. From the network analysis, Community 3 exhibits high centrality values (Degree, Closeness and Betweenness Centrality), as well as high In-Strength, indicating a prominent role in the synchronization and reception of drought signals across the basin. Interestingly, the nodes exhibiting the highest centrality in the undirected network — typically interpreted as influential or well-connected-appear in the directed network as terminal convergence points of drought propagation. This counterintuitive configuration reveals the existence of systemic “sinks” within the basin: areas that are not primary drivers of drought onset, yet function as critical endpoints where propagation patterns consistently culminate. Their centrality arises not from initiating propagation, but from their persistent role as hubs of synchronization and accumulation.

Community 2 spans the central portion of the basin and includes areas with transitional topographic and climatic characteristics. These regions do not exhibit the persistent orographic effects seen in the Alpine north, nor the relative stability of the eastern lowlands. As a result, they experience a wider range of drought conditions, both in terms of frequency and intensity, reflecting the influence of more variable and localized weather dynamics. From the network analysis, Community 2 displays intermediate centrality values across all metrics (DC, BC, CC), suggesting a balanced position within the system-neither a dominant source nor a major receiver of drought signals. This translates into less defined propagation pathways, consistent with the observed variability in drought behavior.

Overall, a speculative explanation of these findings can be proposed as follows: precipitation patterns (and consequently, precipitation deficits) in the Po Basin are strongly influenced by orography. The Alps to the north and the northern Apennines to the south act both as zones where precipitation is intensified-due to orographic reinforcement-and as natural barriers that alter the trajectory and intensity of dominant meteorological disturbances. The interplay between the major meteorological disturbances and this orography shapes the general distribution of precipitation and influences the emergence of drought conditions in different regions of the basin. This interplay gives rise to the regionalization illustrated by the broader patterns observed in Fig. 2. Specifically, the direction and intensity of atmospheric disturbances influence both the rainfall regime over these orographic barriers and the extent to which moisture-laden systems can penetrate downstream into the basin. This framework allows the identification of dominant meteorological configurations that offer a plausible explanation for the spatial distribution of drought conditions across the Po River Basin. While these represent the most recurrent patterns, they do not exclude the occurrence of less frequent setups or localized anomalies. One common configuration involves northwesterly Atlantic disturbances, which frequently impact the outer and northern slopes of the Alps, releasing most of their moisture over the mountains before reaching the Po Valley. This process frequently induces a foehn effect, whereby dry downslope winds significantly reduce precipitation in the lowlands south of the Alps. When such disturbances are weaker, they may be entirely blocked by the Alpine barrier. In these cases, significant precipitation deficits can develop in the Alpine region (Community 1), leading to prolonged dry periods, while the Po Valley receives little or no rainfall. This mechanism may explain why the Alps, despite being among the wettest areas of the basin, are also susceptible to persistent droughts when regular precipitation inputs are disrupted. Another relevant pattern involves disturbances from the northeast, which, although less frequent, can have a pronounced impact — particularly during the winter season. Cold air masses originating from Eastern Europe often gain moisture over the Adriatic Sea, leading to snowfall across the basin, including at relatively low altitudes. A third synoptic pattern involves cyclogenesis over the Ligurian Sea, generating low-pressure systems that can bring intense precipitation to the western portion of the basin. Depending on the broader atmospheric configuration, these systems may extend beyond the Apennines (i.e., beyond Community 2), reaching the eastern and southeastern sectors. However, when the moist airflow is blocked by the Apennine ridge, precipitation tends to remain confined to the coastal and foothill areas, failing to reach the central Po Valley.

## Conclusion

By applying complex network analysis to study the onset and propagation of drought events, we can identify regional patterns (clusters) and pathways of propagation that are driven by physical processes but would typically be difficult to detect. In our analysis, these patterns are significantly influenced by natural orographic barriers, which modulate the impact of dominant meteorological disturbances affecting the region, primarily originating from the north and east. It is crucial to distinguish that drought refers to a sustained lack of precipitation, and not merely to fluctuations or patterns in rainfall. As a result, weaker meteorological disturbances can create precipitation deficits over the Alps, while those smaller disturbances struggle to cross the orographic barrier and reach the valley — this leads to a “propagation” of precipitation deficits. These results emphasize the unique capability of network-based approaches to uncover interdependencies and synchronization patterns that traditional statistical methods, such as those used in Fig. 2, often overlook. Furthermore, methods like Peaks Over Threshold (POT) and spatiotemporal techniques like Empirical Orthogonal Functions (EOFs) do not adequately capture the temporal dynamics of individual drought events. By understanding drought dynamics as the onset locations and propagation directions, together with an understanding of the underlying physical phenomena, this framework supports anticipatory strategies for drought management.

Prioritizing regions for resource allocation and the development of early warning systems could significantly enhance resilience to cascading drought impacts. As Italy’s primary water resource, the Po River Basin is particularly vulnerable due to its role in sustaining agricultural, industrial, and tourism activities, which collectively account for approximately 40% of the country’s GDP. Targeted mitigation strategies, such as strengthening water infrastructure in highly connected regions or implementing conservation measures in areas with intense agricultural and hydrological activity, could help safeguard economic and food security in this critical region. By capturing local and systemic interconnections, this study provides a robust foundation for predicting future drought scenarios as climate change accelerates. Expanding this framework to include agricultural and hydrological droughts would further enhance our understanding of drought resilience, particularly in regions heavily reliant on water resources, such as the Po River Basin.

## Materials and methods

**Fig. 11:**
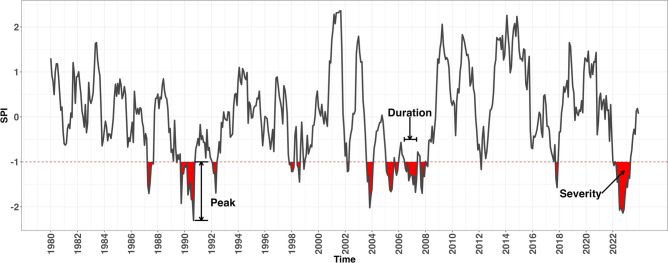
SPI-12 time series of Po River Basin with a schematic representation of drought event characteristics: Peak, Duration and Severity.

This study employs both undirected and directed complex networks to analyze drought synchronization in the Po River Basin. Networks are constructed based on statistical relationships in the 12-month Standardized Precipitation Index (SPI-12) across various spatial locations, and their topological properties are analyzed to understand the spatiotemporal dynamics of drought events. To broaden our analysis, we include additional geographic regions, enabling the exploration of climate interactions that may influence drought patterns within the basin. This approach provides insights into how drought events are shaped by both local factors and broader atmospheric patterns^43,44^. Precipitation data were obtained from the ERA5 dataset^31^, a global atmospheric reanalysis product developed by the European Centre for Medium-Range Weather Forecasts (ECMWF) and made available through the C3S Climate Data Store^45^, which combines model simulations with observational data to provide a consistent climate record. For this study, we obtained hourly weather data spanning from 1980 to 2023 at a spatial resolution of $0.25^{\circ } \times 0.25^{\circ }$ on the WGS84 geoid, covering the area between $43^{\circ }{-}49^{\circ }$N latitude and $4^{\circ }{-}19^{\circ }$E longitude, fully encompassing the Po River Basin. Monthly precipitation (P) was derived by aggregating hourly data from January 1980 to December 2023, producing a continuous time series of 528 monthly entries for each of the 888 grid points in the study area. To assess drought conditions, we applied the SPI at a 12-month timescale (SPI-12), based on methodologies by Edwards and McKee (1997)^24^ and refined by Guttman (1999)^46^. The 12-month aggregation period was chosen to eliminate the effects of seasonality. This approach ensures that the identified droughts are not merely moderate, short-term summer phenomena, but rather significant and persistent events. Their emergence implies a duration of at least one year, emphasizing their hydrological relevance due to the potential impact on overall water availability at the river basin scale. SPI is calculated by fitting a Gamma distribution to precipitation data from 1980 to 2023, which is then transformed into a standard normal distribution to allow for comparative analysis across different locations and timescales. A drought event is defined as any period when SPI-12 falls below -1.0 for at least three consecutive months. Each event is characterized by its duration (the number of consecutive months with SPI-12 below -1.0), severity (the sum of SPI-12 values during the event), and peak (the lowest SPI-12 value within the event). For clarity, Fig. 11 illustrates the time series of SPI-12 values calculated from the total precipitation averaged over the basin.

Furthermore, the computation of drought features is extended to the entire study area, enabling the identification of hotspots at the spatial scale. In this framework, duration is defined as the average length of drought events at each location, severity as the cumulative sum of SPI-12 values associated with drought events over the time series, and peak as the minimum SPI-12 value recorded during these events. The results are detailed in the Results section.

### The Po River Basin

**Fig. 12:**
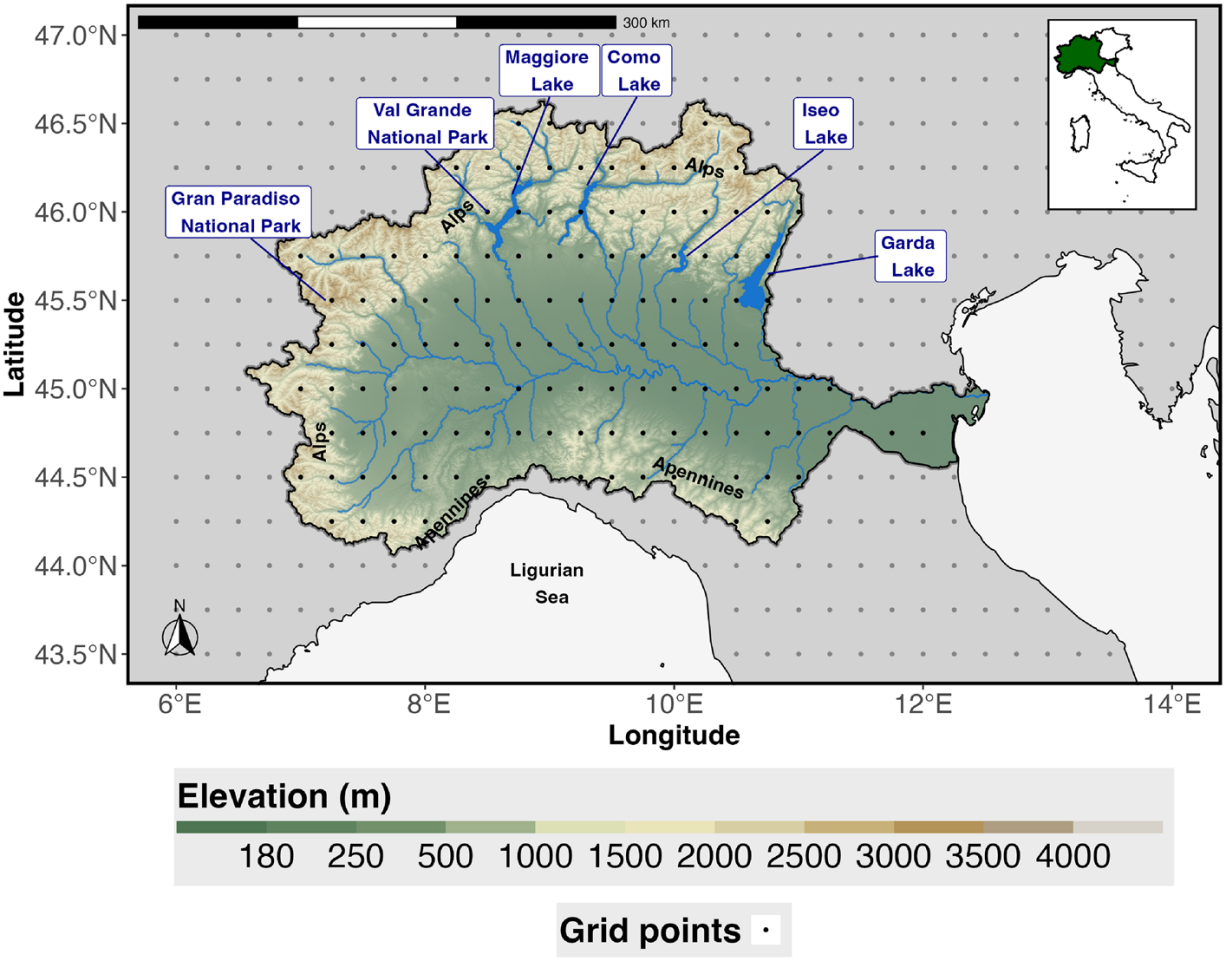
Map of hydro-graphic and topographic Po river basin along with the ERA5 grid points.

The Po is Italy’s largest and longest river, flowing through the northern regions and encompassing a hydrographic basin of approximately 71,000 km^2^ (Fig. 12). This basin, known as the Po Valley, is a low-lying area traversed by numerous tributaries, with sources in the Alps to the north and the Apennines to the south. Figure 12 shows the cell centers of the 0.25 x 0.25 climatic data grid along with a map of the hydrographic and topographic features of the Po River Basin.

**Fig. 13:**
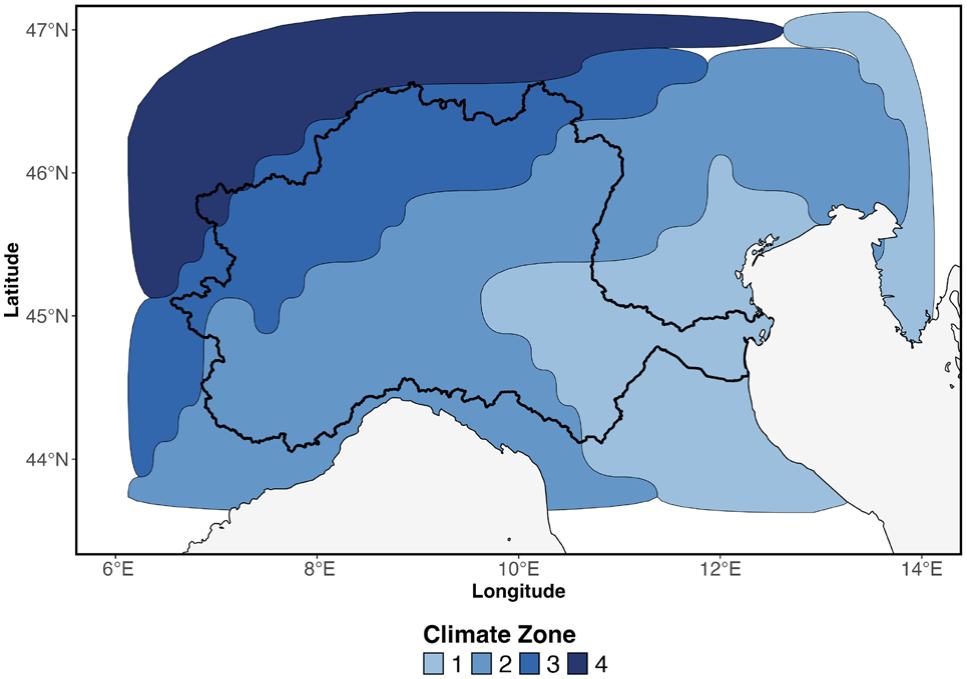
Climate zones of precipitation.

The Po Valley region is characterized by four distinct climate zones based on precipitation (Fig. 13). These climate zones were identified using a k-means clustering approach on the loadings of the first three Empirical Orthogonal Functions (EOFs) of the 12-month Standardized Precipitation Index (SPI-12), which explain 38.4%, 19.4%, and 11.9% of the total variance, respectively. Specifically, the k-means method was executed using the Lloyd algorithm with 1000 iterations and setting the number of clusters to four^47^. Subsequently, the yearly cumulative precipitation from the ERA5 dataset for the period of 1981-2023 was averaged, resulting in the mean yearly precipitation for each climate zone, as detailed in Table 2.

**Table 2:** Descriptive statistics of yearly cumulated precipitation for the identified 4 climate zones (mm).

Statistic	Zone 1	Zone 2	Zone 3	Zone 4
25%	783.6	1054.4	1178.2	1454.7
Median	906.8	1235.2	1422.7	1645.6
75%	1106.4	1407.6	1750.8	1825.7

In recent years, the Po River has experienced several severe drought events, most notably the hydrological drought of summer 2022, which drew international media attention, as highlighted by Montanari et al. (2023)^19,20^. During this period, hundreds of towns in Northern Italy faced water restrictions, and multiple regions declared states of emergency^48,49^.

The impact of extreme weather events in the Po Valley is significant, given the basin’s extensive role in Italy’s national hydrographic network. It covers approximately 23% of the country’s territory and serves around 17 million residents. Economically, the Po Valley is crucial to Italy, contributing 35% of the national agricultural output, 55% of livestock production, and 48% of hydropower generation^50^.

### Spatiotemporal synchronization of drought events

Event Synchronization (ES) was introduced in a seminal paper of 2002 [see^51^ for details], and further redefined in a successive paper of 2010 [see^52^]. ES offers a novel approach to analyzing the relationships between extreme events across different regions. Unlike traditional statistical methods, which often rely on data conforming to specific probability distributions^53,54^, ES emphasizes the frequency with which extreme events from two locations occur close together in time, accommodating minor differences in their timing. Its key advantage lies in its flexibility, as it can be applied to diverse datasets without the need for them to follow predefined distributions. ES is particularly effective in identifying the precise moments when extreme events occur simultaneously at two locations while disregarding periods without events. This specific focus is particularly helpful when investigating irregular occurrences, like extreme weather events, where timing is uncertain and conventional approaches frequently fall short of offering reliable findings^55^.

Building on the strengths of ES, we represent each grid point in the study area as a node within our complex system. Connections, or edges, between these nodes indicate the synchronization of drought events across locations. ES quantifies this synchronization by counting the occurrences of drought onsets at two grid points within a specified time window, allowing dynamic delays between events.

To illustrate this, consider two grid locations $i$ and $j$. Let $t^l_i$ represent the time when a drought event $l$ begins at location $i$, and $t^m_j$ represent the time when event $m$ begins at location $j$. Here, $n_i$ and $n_j$ denote the total number of such events at locations $i$ and $j$, respectively. The dynamic time lag $\tau ^{lm}_{ij}$ between these two events is calculated as:

1 $$\begin{aligned} \tau ^{lm}_{ij} = \min \left\{ \frac{t^{l+1}_i - t^l_i}{2}, \frac{t^l_i - t^{l-1}_i}{2}, \frac{t^{m+1}_j - t^m_j}{2}, \frac{t^m_j - t^{m-1}_j}{2} \right\} \end{aligned}$$

This equation defines the minimum acceptable time lag between drought events at two grid points for synchronization. By taking the minimum of the time intervals between consecutive events at each location and dividing it by two, the method avoids double-counting and ensures that only events occurring within a close temporal window are considered synchronized. A maximum delay of three months ($\tau _{{\max}} = 3$) is set between events at two grid points to avoid excessively long dynamic delays^29,55,56^. Thus, if the time lag between events $t^l_i$ at grid $i$ and $t^m_j$ at grid $j$ falls within the range $(0, \tau ^{lm}_{ij}) \cap (0, \tau _{{\max}})$, the events $l$ and $m$ are considered synchronized.

Using this definition of event synchronization, we calculate the synchronization strength between any two event series, accounting for the order of event occurrences when necessary. This strength serves as the basis for constructing either undirected or directed networks, depending on whether the directionality of event synchronization is included.

### Construction of the undirected network

The undirected network is constructed from the synchronization strength matrix, which captures the connections between grid points without assuming a specific direction. This network provides a framework for identifying the spatial extent and characteristics of synchronized drought events, facilitating further analyses such as regionalization^57^.

The synchronization strength between two grid points $i$ and $j$ is mathematically expressed as:

2 $$\begin{aligned} Q_{ij} = \frac{ES(i|j) + ES(j|i)}{\sqrt{n_i n_j}} \end{aligned}$$

where $ES(i|j)$ is the sum of the synchronization instances where events at grid $i$ follow those at grid $j$, and $ES(j|i)$ is the reverse. As above, $n_i$ and $n_j$ denote the total number of such events at locations $i$ and $j$, respectively. The numerator is calculated using:

3 $$\begin{aligned} S_{ij} = {\left\{ \begin{array}{ll} 1 & {\text{if}}\; 0< t^l_i - t^m_j< \tau ^{lm}_{ij} {\text { and }} 0< t^l_i - t^m_j < \tau _{{\max}} \\ 0.5 & {\text{if}}\; t^l_i = t^m_j \\ 0 & {\text{otherwise}} \end{array}\right. } \end{aligned}$$

The matrix $Q_{ij}$ is normalized between 0 and 1, where $Q_{ij} = 1$ indicates full synchronization of events between grid points $i$ and $j$, and $Q_{ij} = 0$ indicates no synchronization. The matrix $Q$ is symmetric, meaning $Q_{ij} = Q_{ji}$, which is ideal for spatial localization analysis.

To minimize redundancy, only the top 5% of synchronization values are preserved, with the corresponding grid points considered connected. This approach ensures that only statistically significant links are included in the network, providing a robust representation of the most critical connections. The 95th percentile of all non-zero synchronization values, denoted as $\theta$, is used to convert the synchronization strength matrix into a binary adjacency matrix:

4 $$\begin{aligned} A_Q = {\left\{ \begin{array}{ll} 1 & {\text{if}}\; Q_{ij} > \theta {\text { and }} i \ne j \\ 0 & {\text{otherwise}} \end{array}\right. } \end{aligned}$$

**Fig. 14:**
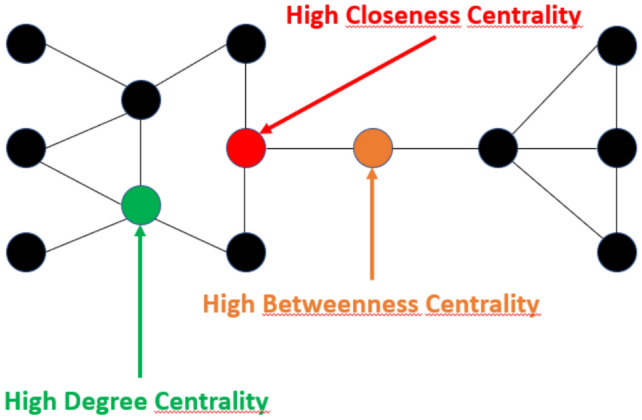
Degree Centrality (DC), Closeness Centrality (CC) and Betweenness Centrality (BC) illustration.

The resulting adjacency matrix $A_Q$ is symmetric. From the undirected network, we derive several network metrics, including Degree Centrality (DC), Closeness Centrality (CC), and Betweenness Centrality (BC). Degree Centrality (DC) measures the number of neighboring grid areas that experience drought occurrences simultaneously, with a lag of 1-3 months. A higher DC indicates that a location plays a key role in synchronizing drought conditions across neighboring regions, acting as a focal point for the spatial co-evolution of drought events over time and thus identifies homogeneous areas.The transition from high DC to low DC areas marks the boundaries between regions highly affected by drought synchronization and those less influenced by synchronized drought dynamics. These transition boundaries are due to the presence of different seasonal precipitation regimes which alter the possibility of having a reduced amount of precipitation at annual time scale. Closeness Centrality (CC) indicates fast-track propagation pathways from a node to all other nodes in the network. In the context of drought events, a higher CC suggests that the location is located on a pathway where drought can be rapidly transmitted to or receive from other synchronized areas in the whole network. Such behavior makes areas with high CC critical, and vulnerable to drought propagation since they represent grid points with a higher global proximity in the entire network considered. Betweenness Centrality (BC) captures the role of a node as a critical bridge in the network, influencing the pathways through which drought events propagate. A higher BC represents a physical route, allowing these areas to act as pivotal junctions in the propagation network. Locations with high BC play a crucial role in the early emergence and large-scale propagation of drought events across the network. The following Fig. 14 visually illustrates each centrality concept.

This network-based framework allows us to identify key regions that influence the spatial spread and co-evolution of drought events within the study area. To evaluate the structural significance of the observed synchronization patterns, we analyzed the distribution of three centrality measures-Degree, Closeness, and Betweenness-using the Empirical Cumulative Distribution Function (ECDF). These metrics capture different aspects of nodal relevance in the context of drought synchronization: local connectivity intensity (Degree), proximity to other nodes in terms of synchronized drought onset (Closeness), and the role in connecting distinct regions of the network (Betweenness). To assess whether these patterns could emerge from random processes, we compared the empirical centrality distributions to those obtained from simulated networks. Specifically, we generated ensembles of 1,000 networks for each of three canonical models-Random (Erdős-Rényi), Small-World (Watts-Strogatz), and Scale-Free (Barabási-Albert)-each preserving the number of nodes (1,525) and edges (58,740) of the observed drought synchronization network. For each simulation, centrality values were computed and normalized using min-max scaling. To further explore the structural organization of drought synchronization across the Po River Basin, we applied the Louvain community detection algorithm to the empirical synchronization network. This method is widely used for identifying modular structures in large networks by optimizing modularity, a measure of how well the network is partitioned into internally cohesive communities with relatively few connections between them^35^. The algorithm proceeds in two main phases: a local optimization step, where each node is initially assigned to its own community and then iteratively moved to neighboring communities if doing so increases modularity; and a hierarchical aggregation step, in which the resulting communities are collapsed into super-nodes to form a new network^37,58^. This process is repeated iteratively until no further improvement in modularity is possible, yielding a final, hierarchical structure that captures the mesoscale organization of the network^35^. In the context of drought synchronization, the resulting communities reveal spatial clusters of regions that tend to experience temporally aligned drought conditions. We conducted 1,000 separate Louvain algorithm runs to evaluate the reliability of the identified modular structure. To assess the consistency of the generated partitions, we calculated the Normalized Mutual Information (NMI) score for every pair of runs^36^. Despite the Louvain method’s intrinsic stochasticity, the average NMI score of 0.88 showed an important level of stability across runs. This supports the validity of the modular decomposition in capturing enduring patterns of drought synchronization across the basin by confirming the robustness and reproducibility of the major community structure, which consists of three prominent clusters.

### Construction of the directed network

The directed network enables us to not only identify where synchronization occurs, but also determine the direction of drought propagation. To achieve this, we calculate the in-strenght and out-strenght centrality for each node (grid point). In-degree centrality measures how frequently a node is synchronized with events in other nodes, indicating how often it is influenced by droughts started elsewhere. In other words these areas are where drought propagation ends. Conversely, out-strenght centrality measures how often a node initiates drought events which then synchronize with other nodes, revealing its role as a source of drought propagation or where drought events start their propagation.

Mathematically, the synchronization strength for the directed network between grid points $i$ and $j$ can be expressed as $ED_{ij}$, which represents the relative frequency of drought events at grid $i$ preceding those at grid $j$:

5 $$\begin{aligned} ED_{ij} = \frac{\sum _{l=1}^{n_i} \sum _{m=1}^{n_j} D_{ij}^{lm}}{n_i} \end{aligned}$$

with $n_i$ and $n_j$ denote the total number of such events at locations $i$ and $j$, respectively and where

6 $$\begin{aligned} D_{ij}^{lm} = {\left\{ \begin{array}{ll} 1 & {\text{if}}\; 0< t^m_j - t^l_i< \tau ^{lm}_{ij} {\text { and }} 0< t^m_j - t^l_i < \tau _{{\max}} \\ 0 & {\text {otherwise}} \end{array}\right. } \end{aligned}$$

In this equation, $D_{ij}^{lm}$ indicates whether the drought event $l$ at grid $i$ and the event $m$ at grid $j$ are synchronized, with event $l$ occurring before event $m$. Importantly, to focus on true propagation patterns, cases where events at the two grids occur simultaneously are excluded from the analysis. $ED_{ij}$ thus reflects the likelihood of a drought event at grid $i$ propagating to grid $j$, serving as a measure of directional synchronization strength. Similarly, $ED_{ji}$ is calculated as:

7 $$\begin{aligned} ED_{ji} = \frac{\sum _{m=1}^{n_j} \sum _{l=1}^{n_i} D_{ji}^{ml}}{n_j} \end{aligned}$$

This metric represents the potential for a drought event at grid $j$ to propagate to grid $i$, capturing the reverse directional relationship.

To retain only the most significant directional relationships, a threshold based on the 95th percentile of all synchronization strength values is applied. Let $\delta$ represent this threshold. The delay strength matrix $ED$ is then converted into the directed adjacency matrix $C$ as follows:

8 $$\begin{aligned} C_{ij} = {\left\{ \begin{array}{ll} ED_{ij} & {\text{if}}\; ED_{ij} > \delta {\text { and }} i \ne j \\ 0 & {\text{otherwise}} \end{array}\right. } \end{aligned}$$

The directed adjacency matrix $C$ is asymmetric, reflecting the unidirectional nature of connections between nodes and forming the basis for constructing a directed complex network. This framework allows for a precise analysis of the directional propagation of drought events. From the directed network, we derive metrics such as strength (total propagation into or out of a node) and dominant orientation (primary direction of propagation) to characterize drought propagation. In this context, in-strength represents the frequency and intensity with which a location receives drought signals from other areas, identifying regions that are more vulnerable to incoming drought conditions. Conversely, out-strength captures how frequently a location initiates drought events that propagate outward, highlighting areas that act as primary sources of drought spread. These metrics provide a comprehensive view of the spatial-temporal dynamics of drought, identifying both source regions that influence broader areas and hydrological hot-spot hubs where drought propagation converges.

## Data Availability

The data and codes used in this study are available at the following link: https://github.com/AntZin84/PO_river_paper
